# Perceptions and practices of general practitioners on providing oral health care to people with diabetes - a qualitative study

**DOI:** 10.1186/s12875-020-1102-9

**Published:** 2020-02-13

**Authors:** Prakash Poudel, Rhonda Griffiths, Vincent W. Wong, Amit Arora, Jeff R. Flack, Chee L. Khoo, Ajesh George

**Affiliations:** 1 Centre for Oral Health Outcomes and Research Translation (COHORT), Liverpool, New South Wales (NSW) 2170 Australia; 2 School of Nursing & Midwifery, Western Sydney University, Campbelltown, NSW 2560 Australia; 3 South Western Sydney Local Health District, Liverpool, NSW 2170 Australia; 4 Ingham Institute for Applied Medical Research, Liverpool, NSW 2170 Australia; 5 Faculty of Medicine, University of New South Wales, Kensington, NSW 2052 Australia; 6 School of Health Sciences, Western Sydney University Campbelltown Campus, Campbelltown, NSW 2751 Australia; 7 Translational Health Research Institute, Western Sydney University, Penrith, NSW 2751 Australia; 8 Discipline of Child and Adolescent Health, Sydney Medical School, Faculty of Medicine and Health, The University of Sydney, Westmead, NSW 2145 Australia; 9 Oral Health Services, Sydney Local Health District and Sydney Dental Hospital, NSW Health, Surry Hills, NSW 2010 Australia; 10 Diabetes Centre Bankstown-Lidcombe Hospital, Bankstown, NSW 2200 Australia; 11 School of Medicine, Western Sydney University, Campbelltown, NSW 2560 Australia; 12 Health focus Family Practice, Ingleburn, NSW 2565 Australia; 13 University of Sydney, Camperdown, NSW 2050 Australia

**Keywords:** Diabetes mellitus, Oral health care, Diabetes care provider, Model of care, General practitioner

## Abstract

**Background:**

Poorly controlled diabetes leads to multiple complications including oral health problems. General practitioners (GPs) are at the forefront of management of chronic diseases in primary health care. Diabetes guidelines encourage a proactive role for GPs in oral health complications management in people with diabetes, yet little is known about this area of care. This study aimed to explore current practices, perceptions and barriers of GPs towards oral health care for people with diabetes.

**Methods:**

We employed a qualitative research method utilising telephone interviews. Purposive and snowball sampling were used to recruit 12 GPs from Greater Sydney region. A thematic analysis involving an inductive approach was used to identify and analyse contextual patterns and themes.

**Results:**

A majority of participants were males (*n* = 10), working in group practices (*n* = 11) with a mean ± SD age of 55 ± 11.4 years and 25 ± 13.6 years work experience. Three major themes emerged: oral health care practices in general practice settings; barriers and enablers to oral health care; and role of diabetes care providers in promoting oral health.

Most GPs acknowledged the importance of oral health care for people with diabetes, identifying their compromised immune capacity and greater risks of infections as risk factors. GPs reported 20–30% of their patients having oral health problems, however their current oral health care practices relating to education, risk assessment and referrals were reported as very limited.

GPs identified several barriers including time constraints, absence of referral pathways, and limited knowledge and training in promoting oral health care. They also reported patient barriers including oral health care costs and lower oral health awareness. GPs perceived that resources such as education/training, a standardised assessment tool and patient education materials could support them in promoting oral health care. GPs also perceived that other diabetes care providers such as diabetes educators could play an important role in promoting oral health.

**Conclusions:**

Despite current recommendations, GPs’ current oral health care practices among people with diabetes are limited. Further strategies including capacity building GPs by developing appropriate oral health training programs and simple risk assessment tools along with accessible referral pathways are needed to address the current barriers.

## Background

Diabetes is a major and growing health problem worldwide. Diabetes caused an estimated 1.6 million deaths and was the seventh leading cause of death in 2016 [1]. In 2015, diabetes affected around 1.2 million (6%) Australians and caused over 16,000 (10%) deaths in 2015–2016 [2]. Due to the complexity of the disease, people living with diabetes require a systematic, ongoing, and organised care plan involving a multidisciplinary health care team [3], which usually involves general practitioner, specialist physician (endocrinologist), diabetes educators (nurses), dietitian, podiatrist, exercise physiologist, and optometrist [4]. In Australia, as in several other countries such as the United States of America, New Zealand, Canada and Singapore [5], general practitioners (GPs) are at the front line for the management of chronic diseases including diabetes, in primary health care and working in collaborative care arrangements [6, 7]. Primary health care services in Australia provide treatment services to patients who are not admitted to the hospital system while specialist care at public hospital provides complex care for situations such as metabolic emergencies, acute cardiovascular disease and kidney failure [6].

Poorly managed diabetes can lead to multiple complications including coronary artery disease, stroke, kidney failure, limb amputation, and blindness [7]. Diabetes is also associated with dental problems [8] and in recent years significant attention has been given toward this association and its implication for people living with diabetes. Studies suggest that people with suboptimal control of diabetes have 2–3 times greater risk of periodontal (gum) disease than people without diabetes [8]. Furthermore, it is also well established that diabetes and periodontal disease have a bidirectional relationship as both negatively affect each other sharing the common pathophysiological mechanisms of infection and inflammation [8]. Therefore, blood glucose control and good oral hygiene are important in preventing and slowing the progression of diabetes complications including periodontal disease. Evidence from systematic reviews of randomised controlled trials also suggest that non-surgical treatment of periodontal disease improves the glycaemic control (HbA1c) of people living with diabetes [9].

Diabetes prevention and management guidelines recommend diabetes care providers incorporate oral health review and referrals into diabetes care [7, 10, 11]. However, there is limited research investigating current practices and perceptions of diabetes care providers in regard to providing oral health care [4]. A few surveys conducted internationally suggest that physicians (general practitioners and specialists) and diabetes educators are less aware about the bidirectional relationship of diabetes and oral health [12–14]. Our previous research conducted among diabetes educators also found that diabetes educators reported a very limited knowledge about oral health and identified this as a major barrier to addressing oral health care of people with diabetes [15]. The limited oral care practices of diabetes care providers also have implications for people living with diabetes who have reported inadequate oral health knowledge, poor oral health attitudes, and lower compliance of recommended oral hygiene behaviours and dental visits [15].

In Australia, people living with diabetes generally visit GPs for diabetes management [16] and therefore encouraging GPs, in line with current clinical guidelines, to incorporate oral health reviews and referral activities as part of diabetes care is pertinent to improving oral health and diabetes outcomes of their patients. Few studies have explored the role of GPs in promoting oral health among general patients in rural and regional Australia and found that they were not very confident in managing oral health complications despite regularly seeing patients with oral health problems [17, 18]. However, to date, no Australian studies have explored this area of general practice from a diabetes care perspective, therefore the aim of this study was to explore current practices, perceptions and barriers of GPs towards oral health care for people living with diabetes.

### Research questions

The following research questions guided this study:

- What are GPs’ perceptions and experiences about oral health care for people living with diabetes?
- How knowledgeable are GPs on the link between diabetes and oral health?
- What are the current oral health care prevention and management practices that GPs provide to people living with diabetes?
- What are perceived barriers and facilitators for GPs to promote oral health care to people living with diabetes?
- What are GPs’ suggestions on the role of diabetes care providers in promoting oral health care to their patients?

## Methods

### Design

The study used a qualitative research design [19] using telephone interviews with GPs responding to open ended questions. A qualitative research method was appropriate as the study intended to explore understanding, experiences and views [20] of the GPs on the research topic. The interviews were conducted over the telephone because of the convenience to recruit time-poor GPs [21] and also considering that they could talk freely and provide detailed information over the telephone [22].

### Sampling and data collection

GPs working in general practices in New South Wales, Australia were eligible to participate. Purposive sampling [23] was used to recruit participants with snowball sampling and word of mouth techniques [23] used to enhance purposive sampling. The advertisement for study recruitment was circulated at various seminars and also through GP newsletters and websites. The flyer and participant information sheet for the study were sent to interested GPs via email to provide further information about the investigators and rationale for the research project. An interview topic guide (See Additional file 1) was developed based on our review of the literature and our previous study conducted with diabetes educators [4, 15] and refined with a multidisciplinary team involved in this research.

The principal researcher (PP, male, MPH, PhD candidate) who was trained in qualitative research and had no prior relationship with any of the participants conducted all the individual interviews. The recruitment process started from March 2018 and was completed in November 2018. A semi-structured interview process was followed by the researcher to ensure that participants spoke freely on each question with the use of open and closed questions and follow-up probes. Furthermore, participants also had an opportunity at the end of the interview to add anything on the questions asked or on the overall research topic [24].

All participants provided informed verbal consent for the telephone interviews which was audio recorded at the beginning of the interviews. Verbal consent was sought due to the time constraints of GPs to undertake face-face interviews and this strategy was approved by the Ethics Committee as part of the approval process. De-briefings were organised with another researcher [AG] [25] to discuss completeness of data and any new areas to explore in subsequent telephone interviews. Recruitment and data collection continued until data saturation [26] when no new information emerged from the interviews. Demographics of the participants including age, gender, years of medical practice, and highest qualification were collected at the end of the interview.

### Data analysis

Interviews were audio recorded and transcribed. Transcripts were checked for accuracy and imported into qualitative data management software (QSR Nvivo 11). The transcripts were individually read and re-read to gain familiarity with the data and to record initial ideas then an overarching coding framework was developed, informed by the interview topic guide. A thematic analysis involving an inductive approach was used to identify and analyse contextual patterns and themes within the data [23]. Two researchers (PP & RG), who were trained in qualitative research, independently coded the transcripts and identified themes and sub-themes from the data. Team meetings were organised to discuss similarities and differences in the themes and interpretations and a consensus was achieved (Table 1).

**Table 1:** Themes and subthemes

Themes	Subthemes
Theme 1: Oral health care in general practice	Perceived importance of oral health care
	Perceived incidence of oral health problems
	Current oral care prevention and management practices
	* Risk assessment*
	* Patient education*
	* Providing Referrals*
Theme 2: Barriers and enablers to oral health care	Barriers for care providers
	* Time*
	* Referral pathways*
	* Oral health knowledge*
	Barriers for patients
	* Cost of oral health care*
	* Limited oral health awareness*
	* Dental fear or anxiety*
	Enablers to oral health care
	* Education/ training*
	* Risk assessment tool*
	* Patient education resources*
Theme 3: Role of diabetes care providers in promoting oral health	Role of GPs
	Role of diabetes educators

### Ethical considerations

This study was a part of a larger study which had received ethics approval from the South Western Sydney Local Health District Research and Ethics Committee (HREC/15/LPool/) and the Human Research Ethics Committee of Western Sydney University (RH12241). The audio recordings and transcripts were stored on a password protected computer as per institutional and ethics committee requirements. Participants were deidentified throughout transcription to ensure the confidentiality and anonymity of the participants. Numeric pseudonyms were used to identify statements from participants (e.g. GP1, GP2).

### Rigor

Several methodological strategies were adopted to enhance the rigor of the study. Telephone interviews were conducted by a researcher trained in qualitative research. Debriefings were organised with another researcher [25] to discuss completeness of data and any new areas to explore in subsequent telephone interviews and continued until data saturation confirmed in the analysis [27, 28]. A professional transcription service was used to improve accuracy of the verbatim transcriptions of the audio recordings. Member checking of the transcripts was not feasible due to the time constraints of participants. Two members (PP & RG) independently checked the data for accuracy and performed the coding. Coding consensus was achieved with the team. Adequate information about the participants, study settings, and data collection are provided in the results and findings are supported by direct quotes of the participants. Reporting of this qualitative research has been undertaken using the Consolidated Criteria for Reporting Qualitative Studies (COREQ): 32- item checklist [See Additional file 2] [29]. The use of these strategies in this study has addressed the criteria for robust qualitative research (credibility, transferability, dependability and confirmability) and ensured the trustworthiness of the research [27, 28, 30].

## Results

Interviews lasted between 9 and 18 min (mean 13.5, SD 2.78). Generally, telephone interviews are shorter than those conducted face-to-face as several factors contribute to the length of an interview such as, the research topic and how much the participant has to say on the research topic or they choose to share, and availability of participant’s time [24]. The thematic analysis of the interview data provided three major themes: Oral health care in general practice settings; Barriers and enablers to oral health care; and Role of diabetes care providers in promoting oral health (Table 1).

### Characteristics of participants

Twelve GPs working in general practices across Sydney South West (*n* = 5), West (*n* = 3) and North (*n* = 2), and the Southern Highlands (n = 2) in New South Wales, Australia participated in this qualitative study. Of the 12 GPs, 11 were practicing in group practice settings, 10 were male, and nine were over 40 years old (range 37–70 years old). The mean time that the GPs had worked in their practice was 25 years (range 5–42 years). Half (*n* = 6) of the GPs had a medical fellowship qualification such as Fellowship of the Royal Australian College of General Practitioners (FRACGP), followed by undergraduate (*n* = 4), and postgraduate (Masters) degrees (*n* = 2).

### Theme 1: oral health care in general practice

There was general consensus among the GPs that oral health care is important for people living with diabetes.

#### Perceived importance of oral health care

Most of the GPs highlighted the significance of oral health describing the compromised immunity of people living with diabetes and resulting risk of infections including oral health problems. As one GP mentioned:

*Yeah. As you know oral health is very important, particularly in the patient with the chronic medical conditions … for example, if someone has diabetes and is not having good oral health, because diabetic people are not having very good immune system and they have high sugar in their blood, so they become susceptible for infection. Oral health could become a serious issue with these people. (GP-8, 66 years old).*

Some of the GPs also recognised the relevance of promoting oral health care in people living with diabetes and expressed willingness to play an active role in this area as part of clinical practice:

*I think it’s good that somebody has taken an interest [in oral health care]. I would definitely like to learn more and being a proactive GP I would definitely be interested in more activities [training and workshops]. (GP-3, 55 years old).*

#### Perceived incidence of oral health problems

While exploring the incidence of oral health problems in patient with diabetes, most GPs reported seeing between 20 to 30% of patients with diabetes having oral health problems:

*Okay, I would probably say about - from my cohort of patients, it’s probably about - out of 10, maybe 2 to three the most. [would have dental problems] it’s very broad I’m answering that because obviously I don’t have finer details (GP-3, 55 years old).*

However, one GP working in Western Sydney mentioned that *“seven out of 10 patients would have issues [oral health problems]” (GP-6, 39 years old)*. In contrast, GPs working in Northern Sydney reported seeing very few patients with oral health problems:

*My practice is sort of a middle-class area and there are very, very few people who have bad oral health in my practice. (GP-4, 70 years old).*

#### Current oral care prevention and management practices

Although most of the GPs highlighted the importance of oral health care for people living with diabetes, their current practices in this area were found to be very limited. The discussion around current oral health care practices focussed on risk assessments, patient education, and providing referrals to a dentist.

##### Risk assessment

Most of the GPs reported that they never assessed their patients with diabetes for oral health risks, except when oral health problems were raised by the patient such as, “*patients come in saying I can’t eat because I have rotten tooth” (GP-10, 48 years old)*.

Another GP mentioned:

*So, if a patient complains of oral health issues, then I would certainly make sure that the patient’s glycaemic control is good. If that is good then you will want to know - basically take the history as to when these oral health issues started and at the time you proceed to - you can backtrack to see whether you think or suspect that at the time their oral health problems began, the glycaemic control wasn’t so good. (GP-5, 67 years old).*

In one of the large GP practices, oral health was included in a care plan for the people living with diabetes and practice nurses inquired about oral health as part of the care team approach adopted:

*We don’t do a formal screening process, but we certainly ask them how their teeth are... All of my patients [with diabetes] get seen four times a year and have a care plan review four times a year. That’s actually one of the things that the practice nurse who helps me do the care plans has on their list. It’s actually on their form in terms of asking questions about a patient’s sleep, a patient’s exercise and they ask about their dental health and their last trip to the dentist (GP-11, 61 years old).*

##### Patient education

Most GPs rarely provided oral health education to their patients or discussed oral health issues.

*I don’t bring it up, no. I’m focused on internal medicine and oral health, I leave to the dentist… my focus of practice is not on oral health (GP-4, 70 years old).*

Furthermore, one participant explained the reason for not educating patients about risks of oral health problems was that “…*it [oral health] has not really been emphasised or publicised and GPs are not being advised of it [to educate]. It is something that only touches on if the patient raises it” (GP-5, 67 years old).*

##### Providing referrals

Most GPs reported they would suggest patients see a dentist if they are experiencing any dental problems:

*We see people with diabetic dental abscesses, dental infections. So we treat them with antibiotics and refer - advise them to go and see the local dentist (GP-2, 60 years old).*

However, all of the GPs agreed that there is a lack of formal systems in place to provide specific dental referrals. As one participant mentioned “it is *not a formal referral. We just say look go and see your favourite dentist” (GP-11, 61 years old).*

### Theme 2: barriers and enablers to oral health care

GPs reported several barriers to promoting oral health care from both the care providers and patients’ perspective. Major barriers for care providers included lack of time and absence of dental referral pathways, while cost of dental care and limited awareness of the link between diabetes and oral health were the barriers for patients.

#### Barriers for care providers

##### Time

Most GPs reported time constraints as a major barrier for not including oral health as part of their routine care for patients with diabetes. As one participant mentioned “…. *time restriction, you’re focusing more on medication, very rare I talk to patients about dental” (GP-12, 40 years old).*

*Unfortunately, we don’t have time. Apart from a simple, just a glance at the teeth and then request them to go to dentist” (GP-9, 57 years old).*

However, some GPs pointed out that they do “…*invest a lot of time into chronic health conditions so time is not an issue [for oral health] (GP-6, 39 years old).* As one GP expressed:

*Well a brief inspection of the mouth only takes 15 or 20 s. Do you see any rotten teeth? Do you see [caries]? Do you see severe gingivitis? I don’t think there’s any reason why a GP can’t do that [discuss oral health]. It doesn’t take long, but it’s one more burden on top of all of the other things that a GP is expected to do. But, for me, it’s part of a standard diabetic check. (GP-11, 61 years old).*

##### Referral pathways

Most of the GPs perceived that most of their patients cannot afford the cost of private dental care and indicated the unavailability of public dental care services to address the dental care need of people the eligible people. They reported unavailability of a referral system to prioritise these patients on these public dental services, as an additional barrier to discussing oral health concerns with their patients. As several GPs highlighted:

*I suppose it’s more to do with the fact that it is a lot of the time not covered by the Medicare Benefit Scheme [Government benefit to GPs]. So that if we do pick something up, what do we do about it? what can the patient do about it in the public system? There isn’t much back up because there’s such a huge queue in the system (GP-2, 60 years old).*

*I don’t think I can even send people to my local hospital, because you’ve got to be in grave health kind of danger before they take you on. That’s unfortunate. (GP-10, 48 years old).*

*Having a well-established network of dental services would be useful. Understanding how the dentist would operate, also having the dentist as part of the team would be useful as well. Like if we think of the diabetic patient. We think of the podiatrist or the kidney specialist, or the cardiologist; well they have an established role. They are - they communicate to us, they recognise their role. Similarly, I think the dentist has to establish their role in that team. (GP-9, 57 years old).*

##### Oral health knowledge

There was divided opinion among participants regarding their knowledge about oral health. Few GPs perceived they had adequate knowledge on this topic and “*don’t think knowledge is a big deal”* (GP-12, 40 years old). Some of the GPs explained the association between diabetes and oral health:

*I’m not speaking for all GPs, but I think most of us the association is with infection. So that’s pretty much how much we know and anything other than that we palm off to the dentist (GP-6, 39 years old).*

*I recognise that a good dentition is necessary for good oral health. A bad dentition can be the source of inflammation. It can increase inflammatory markers in the body. This can be detrimental to the body generally, but also to any other chronic condition that you are trying to manage. So diabetes can be adversely affected by poor dentitions, broadly speaking (GP-9, 57 years old).*

However, a few others perceived they “…*do not have enough knowledge about oral health” (GP- 6, 39 years old)* and this was a barrier in providing oral health care to their patients. None of the GPs had received training on oral health as part of their undergraduate course:

*Generally, we’re poorly educated about teeth. We learn very little about teeth in our undergraduate and postgraduate. We try and get around that by every year, at least, we have one or two registrar training sessions to teach them about dental emergencies. But on the whole, we know very little about teeth and that’s probably the major barrier (GP-11, 61 years old).*

Almost all of the GPs were unaware of the bidirectional link between diabetes and oral health and none were familiar about any diabetes guidelines that address oral health care:

*No, there’s no guidelines that I could think of that would - that actually tells on the area of oral health with diabetics. I don’t think there is. Maybe I’ve missed it. (GP-5, 67 years old).*

#### Barriers for patients

GPs also perceived high cost for dental care, limited awareness about oral health and dental anxiety as barriers for patients and contributed to their poor oral health status and lower dental visits.

##### Cost (finance) of oral health care

The cost of dental care was clearly a major barrier perceived by GPs in accessing oral health care. As one GP stated *“we just say look go and see your favourite dentist. Often the answer is I can’t afford it” (GP-2, 60 years old). A*nother GP recalled his recent experience:

*Because recently I’ve had - this is just an example - a type 1 diabetic who has not been to a dentist in the last eight years purely because he has five children, he doesn’t have private health and he can’t afford it. (GP-6, 39 years old).*

##### Limited oral health awareness

Some GPs also perceived lack of awareness as an additional barrier to patients maintaining their oral health care.

*Education I guess, patients will not have the knowledge of the link between diabetes and oral health” (GP-3, 55 years old).*

##### Dental fear or anxiety

One of the GPs also indicated that patients often perceive fear to see the dentists because of the discomfort or pain on oral health care.

*The other major barrier is the perception of the actual dental management. See, most patients regard going to the dentist as a painful episode. They’re really worried about the pain they may have to endure, so that is also a factor. (GP-9, 57 years old).*

#### Enablers to oral health care

Several enablers to oral health care were also explored with the GPs which included education/training, risk assessment tool and patient education resources.

##### Education/training

Generally, all of the GPs were receptive to education/training on oral health and most believed it would encourage them to provide oral health care to their patients. One GP emphasised the need for *“Education of doctors, education of practice nurses (in oral health). I think they’re the main ones (GP-11, 61 years old).*

*I think that probably more education [oral health] for us [GPs] regarding diabetic oral health is needed and that will actually then [assist] - because I think that’s one area that we’re not really that well alerted to, or educated to, or knowledgeable enough to know and look for things. (GP-5, 67 years old).*

Considering their busy schedule, GPs preferred a short session on oral health *“may be a two-hour session, one-day session may be too much” (GP-7, 61 years old)*, preferably in the evening *“at my age I quite like evening sessions (GP-11,61 years old)* and conducted on online mode *“I’m exceptionally busy in this practice here, but I would be more than happy just to do, if it’s online, like an evening course (GP-8,66 years old).* GPs were also attracted to education sessions that contributed to continuing professional development (CPD) points and a few also suggested that it could be integrated in the diabetes training module:

*The only way to attract GPs to training of some sort is if they get something out of it in terms of…may be CPD points. But… if you did a training module on diabetes and slotted in a presentation on oral health, I would say there’ll be bigger takers than if you just did a course only on oral health in diabetes. But I think this is a big scope for oral health to be put into here [health pathways- it is an online portal for GPs and healthcare professionals which has clinical and referral material for GPs to use on their day-to-day consultation with patients] because this would be the best education. Health Pathways is taking on brilliantly well with registrars and junior doctors and registered nurses in hospitals and in training. (GP 6, 39 years old).*

##### Risk assessment tool

GPs highlighted the need for appropriate assessment tools that could assist screening and risk assessment:

*“A questionnaire would be good, but don’t make it too complicated or long” (GP-10,48 years old).*

##### Patient education resources

All of the GPs wanted to have patient education resources such as brochure:

*“We’d like to get brochure and save the time. We can just give patients and that will - at least remind us to talk about, then after giving a brief discussion we can give it to them” (GP-7, 61 years old).*

### Theme 3: role of diabetes care providers in promoting oral health

#### Role of GPs

Despite time constraints, a majority of GPs perceived they do have some role in promoting oral health.

“…*such a good idea [reviewing oral health] because it’ll trigger us to think about it more at least, more than we should. (GP-1, 37 years old).*

*In a busy practice it can be quite challenging to focus on oral health. But we need to make a point - I do try my best within the restraint” (GP-3, 55 years old).*

One GP though believed that GPs have limited or no role to play in promoting oral health:

*… looking after oral health is such a basic function like going to the toilet and wiping your backside that it’s sort of - it’s a little bit too peripheral for GPs to tell people I think. I mean, public health education maybe if you want pamphlets and all that, but it’s not a big issue as far as I can see. (GP-5, 67 years old).*

#### Role of diabetes educators

Some GPs also acknowledged that other diabetes care providers such as diabetes educators could also play an important role “… *because they actually help to outline some of the things that we GPs don’t have time for” (GP-5, 67 years old)*. However, the capacity of diabetes educators needs to be assessed while scoping this new role as one GP highlighted:

*I’m not sure. Depending on their level of education and their understanding of pathophysiology of the disease. I guess I’m not sure. I guess I’d be more comfortable with a GP talking about it (GP-1, 37 years old).*

## Discussion

This study identified that there is a gap in diabetes guidelines and practices in relation to oral health care of people with diabetes in general practice settings. Overall, GPs acknowledged the importance of oral health care and identified the increased risk of oral health problems, as well as reported frequently seeing oral health problems in people with diabetes. GPs working in South and Western Sydney areas were likely to report seeing more patients with oral health problems than those working in other areas partly because there are significant pockets of disadvantaged and culturally and linguistically diverse (CALD) populations in these areas [31, 32]. It is evident that oral diseases disproportionally affect such communities and are closely linked to socio economic status [32–34]. Studies conducted with rural GPs in Australia also reported oral health problems are common in their patients [17, 18] and reported GPs managing dental problems with antibiotics which is consistent with the practices of GPs reported in our study. However, such practices have often been criticised by dentists as poor management with an inappropriate use of antibiotics [35].

Current practices of GPs in relation to patient education, risk assessment and referrals were reported as very limited which appears consistent with other studies conducted among physicians worldwide [13, 14]. The reason behind why GPs would not address oral health care, unless raised by patients, are multifaceted with some of the reasons being time constraints and lack of referral pathway, as well as limited knowledge and training on oral health. These findings are not new [4] as people living with diabetes usually have multiple medical co-morbidities and GPs may also need to handle other episodic illnesses during their appointment and follow up visits and therefore, oral health may be a lower priority within limited consultation time [14]. One way of addressing time constraints could be to seek assistance from nurses/practice nurses to undertake oral health reviews as part of routine checks as highlighted by one the study participants who was practicing outside of the metropolitan Sydney. This is an area that has potential and should be explored further particularly as studies have shown that patients with diabetes are more likely to visit practice nurses and have a management plan [16]. However, the availability of GP practice nurses in metropolitan areas is limited [36], and needs to be taken into consideration.

Consistent with another study in the UK [37], none of the GPs who participated in this study received any structured oral health training as part of their university curriculum. Similarly, GPs reported very limited knowledge on the bidirectional relationship of diabetes and periodontal diseases. This finding is similar to other studies conducted overseas, where physicians reported they could identify the symptoms of dental problems, however they had limited knowledge or awareness about the reverse effects of periodontal diseases on diabetes [13, 14]. Furthermore, none of the GPs in our study were aware of clinical practice guidelines around diabetes and oral health [7, 10, 11]. Lack of awareness and familiarity are reported as barriers to lower adherence of guidelines [38] therefore, adequate attention needs to be given to encourage GPs to adopt clinical guidelines to underpin best practice. Since GPs were found very receptive to short training or workshops, it is crucial to develop an oral health training program awarding Continuing Professional Development (CPD) points from a professional college, such as the Royal Australian College of General Practitioners. Similar to the findings of our study, other studies also report that health care professional are more attracted to and see the value of CPD activities [39] as GPs are required to meet minimum CPD requirements to maintain their vocational registration [40]. We suggest that the CPD training course should be developed to cater the learning needs of GPs on oral health particularly, in assessment of oral health risks, identifying periodontal diseases and initiating referrals to a dentist. Our previous study with diabetes educators also highlighted that they had very limited knowledge on oral health problems and were unaware about the pathophysiological link of diabetes and oral health. As a result, they were found less confident to promote oral health to their patients [15].

As preferred by a majority of the GPs in our study and considering time constraints which is frequently reported for non-uptake of the free resources [41], it is recommended that the training programs should be delivered using more flexible approaches, preferably online evening sessions as a time-saving method [41] or in a blended learning mode. It is also important that oral heath training is provided at the undergraduate level to ensure new graduate GPs have the basic knowledge in this area and are comfortable in promoting oral health. Unfortunately, as evidenced in the findings, this aspect of training is limited across Australian Universities [42] and is an area that needs to be urgently improved particularly as other professionals like midwifery have successfully integrated oral health into undergraduate programs [43]. Similarly, a short risk assessment tool and a patient education brochure in simple language [15] needs to be developed to assist clinicians to carry out a brief oral health risk assessment and education respectively in general practice. Previous studies have been successful in developing short oral health assessment tools for non-dental professionals to identify other patients at risk of poor oral health [44, 45]. It is equally important that these risk assessments are linked to formalised dental referral pathways. Unfortunately this is another barrier in Australia as dental care is not funded under Medicare, the publicly funded universal health care system [17]. All GPs interviewed in this study also reported the high cost of dental care as an additional obstacle for patients to see a private dentist. Similar findings have been reported worldwide [15]. In Australia around one-third (32%) of people aged 5 years and over avoided or delayed visiting a dentist due to cost in 2013. Furthermore, twice as many people eligible for public dental care (46%) stated they would have difficulty paying for a basic preventive visits than those ineligible for public dental care (22%) and also reported to have higher rates of periodontal disease (33.6%) than those not eligible (19.5%) [46]. In contrast, public oral health services reported to have capacity to provide treatment services for only about 20% of the eligible group and the burden on oral health services is worsened by a significant increase in the number of people waiting to receive dental care, resulting in long waiting times up to 24 months in some cases [47]. Access to oral health care in low income and middle income countries is also an issue and is often reported as unavailable, unaffordable and inappropriate for majority of the population [48]. Therefore, it is crucial to identify and develop a clear referral pathway [15, 17] ensuring an inter-sectorial collaboration, priority access and fee exemption policies [49] to provide dental care for these patients at risk and integrated with primary care services [48]. Additionally, strategies could also be explored to improve access and reduce wait times for public dental services through the use of an alternative dental workforce such as dental therapists, oral health therapists, and dental hygienists. The use of this dental workforce can be a more cost-effective way of providing preventative and non-complex oral health care to children and adults than relying predominantly on dentists [50–53].

Lastly, the findings suggest that diabetes educators could assist in promoting oral health in public diabetes clinics as they are a part of the multidisciplinary diabetes care team [15]. Our previous study also found that diabetes educators overwhelmingly believe and are supportive to the role of promoting oral health, however they expressed concerns that providing oral health information, without knowing whether GPs were going to follow up with it, would be counterintuitive [15]. Therefore, an effective communication amongst diabetes care providers is essential. In general practice settings, practice nurses or diabetes educators could also assist GPs by undertaking the review of annual care including oral health care of the patients, while a brief risk assessment and referral could be done by GPs. Given the time constraints for these busy clinicians, it is of great significance to make oral health review as brief as possible, so that GPs would not perceive it as taking on the workload of dentists and lead to animosity between the discipline of medicine and dentistry [37].

### Limitations

The small number of GPs (*n* = 12) who participated in the study were from four regions in NSW and as such the findings may not be representative of the practices and perceptions of GPs working in other areas or in specific populations. However, considering that there are no other studies conducted on this area in Australia, we believe our findings are valuable and can pave the way for further research to confirm the findings. However, it is also worth noting that our results are consistent with studies conducted in other healthcare settings, thus they do potentially have implications for diabetes care providers worldwide.

### Implication for practice, policy and future research

Our research findings suggest GPs are ill equipped to promote oral health care. There is an important opportunity to identify oral health problems in this at-risk population and improve their oral health care. Several measures are required to design and improve current diabetes clinical practice and patient care to incorporate oral health care. Such improvement requires the development of an evidence-based model of oral health care, including capacity building of GPs, development of resources (risk assessment tool and patent education materials) and identifying a clear referral pathway. Such a model of care could also address the unmet needs of patients with diabetes and improve their oral health care. Adequate consideration should be given to health literacy of patients while developing educational resources such as leaflets. A short risk assessment tool needs to be developed and validated for use by care providers to correctly identify patients at risk for poor oral health. An inter-professional team care approach, integrating with all the diabetes care providers should be promoted and integrated into primary care. Future research should explore the role of nurses/practice nurses in assisting GPs in oral care review of patients.

## Conclusions

Despite recognising the importance of oral health to people living with diabetes, current practices of GPs in relation to oral health education, risk assessment and referral activities were limited. However, GPs were very receptive to promoting oral health care and since they are the primary care providers for people living with diabetes, they should be encouraged and supported to undertake this role considering current barriers. There is an increased attention to encourage non-dental professionals in oral health promotion in various health care settings in Australia [17, 43, 54] and worldwide [55, 56] and research is showing that such programs are acceptable and feasible with the potential for widespread use [43]. Considering such evidence, diabetes care providers should take this opportunity to promote oral health care to their patients. A team care approach, shifting from an isolated dentist centred model of care, is essential to incorporate oral health into routine diabetes care [15, 48].

## Supplementary information

**Additional file 1.** Telephone interview guide for GPs.

**Additional file 2.** Consolidated criteria for reporting qualitative studies (COREQ): 32-item checklist.

## Data Availability

The *file 1* shows the interview guide used for this research. The qualitative data collected and analysed during the current study may available from the corresponding author on reasonable request.

## Abbreviations

CPD: Continuing Professional Development
GPs: General Practitioners
