# Supplementary material for: Perceptions and practices of general practitioners on providing oral health care to people with diabetes - a qualitative study
Source: BMC Fam Pract. 2020 Feb 13;21:34. doi: 10.1186/s12875-020-1102-9 (PMC7020546; doi:10.1186/s12875-020-1102-9)
Supplement: Supplementary file 1 — Additional file 1. Telephone interview guide for GPs. [file 12875_2020_1102_MOESM1_ESM.docx]

Telephone Interview with GPs

*PhD research project/ aims to develop a diabetes oral health program/ interview to know your views/ will be recorded/ confidentiality in relation to data collected for the study and in any reports published/ transcripts from the interview will be coded and any identifying information will be removed/ verbal consent*

1. Could you please elaborate about the importance of oral health care to people with diabetes…?
2. Thinking about the prevalence of oral health problems in people with diabetes at your practice, how many out 10 patients would have oral health problems?
3. Is oral health one of the potential complications you discuss during consultations with people with diabetes?
   1. Could you please elaborate….
      1. do you educate patients about oral health risks?
      2. Do you undertake oral health risk assessment?
      3. Do you provide referrals to the patients with oral health problems?
4. Could you please elaborate about the barriers to promoting oral health among people with diabetes?
   1. For care providers.
   2. For patients (to adopt good oral hygiene behaviours, see dentist…)
5. Thinking about the diabetes clinical practice guidelines, are you aware of any guidelines on oral health care for people with diabetes?
6. Do you have education resources (such as brochures, pamphlets) related to oral health for people with diabetes?
   1. If yes, what sort of resources do you currently have
   2. If no, would you like resources that could assist in promoting oral health? Please elaborate
7. Have you received any education/ training on oral health care for patients with Diabetes?
   1. If yes, where did you find this information. Undergraduate/CDP?
8. Would you be interested to undertake any education/training on oral health (will the CPD course be more attractive?)
   1. If so, are you interested in an evening session, an online program, or half-day weekend seminar?
   2. If no, could you please elaborate…
9. In your opinion do diabetes care providers have important role to promote oral health in routine diabetes care.
   1. If yes, could you please explain who would have the most important role? (Diabetes educators, Endocrinologist, Dietician)
   2. If no, could you please elaborate….
10. Are there any other comments or suggestions you would like to make on this topic?
